# Metabolomic Study to Evaluate the Transformations of Extra-Virgin Olive Oil’s Antioxidant Phytochemicals During In Vitro Gastrointestinal Digestion

**DOI:** 10.3390/antiox9040302

**Published:** 2020-04-06

**Authors:** Gabriele Rocchetti, Biancamaria Senizza, Gianluca Giuberti, Domenico Montesano, Marco Trevisan, Luigi Lucini

**Affiliations:** 1 Department for Sustainable Food Process, Università Cattolica del Sacro Cuore, Via Emilia Parmense 84, 29122 Piacenza, Italy; biancamaria.senizza@unicatt.it (B.S.); gianluca.giuberti@unicatt.it (G.G.); marco.trevisan@unicatt.it (M.T.); luigi.lucini@unicatt.it (L.L.); 2 Department of Pharmaceutical Sciences, Section of Food Science and Nutrition, University of Perugia, via San Costanzo, 06126 Perugia, Italy; domenico.montesano@unipg.it

**Keywords:** EVOO, polyphenols, sterols, foodomics, UHPLC-QTOF, tyrosol derivatives

## Abstract

In this work, different commercial extra-virgin olive oils (EVOO) were subjected to in vitro gastrointestinal digestion and the changes in bioactive compounds were evaluated by ultra-high-pressure liquid chromatography coupled with quadrupole-time-of-flight mass spectrometry, using untargeted metabolomics. As expected, raw EVOO samples were abundant in total sterols (on average: 3007.4 mg equivalents/kg) and tyrosol equivalents (on average: 334.1 mg equivalents/kg). However, the UHPLC-QTOF screening allowed us to annotate 309 compounds, with a large abundance of sterols (219 compounds), followed by polyphenols (67 compounds) and terpenoids. The in vitro gastrointestinal digestion was found to affect the phytochemical composition of the different EVOO samples. In particular, both unsupervised and supervised statistics depicted the modifications of the bioactive profile following gastric and pancreatic phases. Overall, the compounds which resulted as the most affected by the in vitro digestion were flavonoids (cyanidin and luteolin equivalents), whilst relatively high % bioaccessibility values were recorded for tyrosol equivalents during the pancreatic phase (on average, 66%). In this regard, oleuropein-aglycone (i.e., the major phenolic compound in EVOO) was converted to hydroxytyrosol, moving from an average value of 1.3 (prior to the in vitro digestion) up to 9.7 mg equivalents/kg during the pancreatic step. As proposed in the literature, the increase in hydroxytyrosol might be the result of the combined effect of lipase(s) activity and acidic conditions. Taken together, the present findings corroborate the suitability of untargeted metabolomics coupled to in vitro digestion methods to investigate the bioaccessibility of phenolic compounds. In this regard, a significant impact of in vitro gastrointestinal digestion on polyphenolic profiles has been detected, thus suggesting the need to account for actual bioaccessibility values rather than just considering the amounts in the raw commodity.

## 1. Introduction

Olive oil is one of the most important dietary components in the Mediterranean area. Regarding olive oil quality, the International Olive Council has defined different grades according to chemical composition and degree of acidity, with the best brand corresponding to Extra-Virgin Olive Oil (EVOO). EVOO has been widely studied because of its effects in preventing several metabolic diseases and pathologies, such as atherosclerosis, cardiovascular diseases, serum lipoprotein levels, oxidative stress, obesity, type 2 diabetes, inflammation and cancer [1,2,3,4,5]. Overall, the health-promoting effects of EVOO are mainly related to its inherent chemical composition, such as the fatty acid profile [6] rather than other “minor” components (nearly 2% of the total weight). This quantitatively lower fraction includes more than 200 chemically different compounds belonging to aliphatic alcohols, terpenoids, sterols, pigments, volatile compounds and (poly)-phenolic compounds [7,8]. EVOO is reported to contain at least 30 phenolic compounds, including phenolic acids and derivatives, phenolic alcohols, secoiridoids, lignans and flavones [9,10,11].

In particular, tyrosol, hydroxytyrosol, flavonoids (apigenin, luteolin), oleuropein and oleocanthal are among the most characteristic phenolic compounds of olive oil. On average, olive oil consists of 400–500 mg/kg phenolics [12,13]. It is noteworthy that in the last years, great interest has been placed on hydroxytyrosol; in fact, this phenolic compound was entitled by the “health claim” from the European Food Safety Authority, reporting that “the consumption of olive oil rich in polyphenols (hydroxytyrosol, 5 mg/day) contributes to the protection of oxidative damage to lipids in blood” [14]. In addition, different studies focusing on hydroxytyrosol demonstrated several in vitro biological activities exerted by this compound, mainly related to the modulation of gene expression together with potential anticancer activities [15,16]. However, the amount and composition of these compounds in both olives and olive oils clearly result from the complex interactions between several factors, namely, genetic factors (cultivar) and pedoclimatic conditions [17]. Regarding the sterol content found in the unsaponifiable fraction of olive oils, the main phytosterols reported in the literature are β-sitosterol, campesterol and Δ^5^-avenasterol, that are characterized by anti-inflammatory, antibacterial and anti-tumoral activities [18]. Although phenolic compounds of olive oil have demonstrated a relevant activity by several in vitro and animal models, comparatively weaker evidence could be gained by human clinical trials and dietary interventions [19]. One of the possible explanations is that limited information is reported in the literature regarding the modifications of EVOO phytochemicals during the gastrointestinal processes. Overall, the highest plasma concentration of polyphenols and derived metabolites is recorded from 1 to 3 h after ingestion, thus clearly indicating that their major absorption takes place in the small intestine [7]. To date, few studies regarding the impact of a simulated gastrointestinal digestion method on the comprehensive modification of EVOO bioactive compounds have been reported. In particular, previous studies [20,21] showed the stability of both tyrosol and hydroxytyrosol during in vitro simulated digestion, whilst González et al. [22] studied the evolution of the phenolic profile, characterizing olive leaf extract encapsulated by spray-drying, demonstrating an increased phenolic bioaccessibility following encapsulation. In addition, Soler et al. [23] evaluated the bioaccessibility of olive oil phenols, showing a limited metabolism by using Caco-2/Tc7 cells as a model of the human intestinal epithelium. What is even more evident is the lack of comprehensive works based on the combination of in vitro gastrointestinal digestion and untargeted metabolomics to assess the modifications of polyphenols and phytosterols in EVOO during a simulated in vitro digestion process.

Therefore, in this work, simulated static in vitro gastrointestinal digestion followed by untargeted metabolomics was used in order to shed light onto the changes and bioaccessibility of bioactive compounds characterizing five commercial EVOO samples chosen among the most representative cultivars of the Mediterranean basin. In this work, we aimed to achieve a comprehensive compounds profiling, ultra-high-pressure liquid chromatography was coupled with a quadrupole-time-of-flight mass spectrometry (UHPLC-QTOF-MS) and multivariate statistical analysis was applied in order to highlight the compounds undergoing extensive changes during in vitro gastrointestinal digestion.

## 2. Materials and Methods

### 2.1. EVOO Samples and Extraction Step

In this work, five commercial EVOO samples were acquired from local supermarkets (Piacenza, Italy). These EVOO samples were chosen because they are representative of typical olive cultivars spread in the Mediterranean basin, namely Leccino and Frantoio (normally grown in Italy), Picual and Picholine marocaine (characteristic of Spain and Northern Africa) and Kalamon (typical of Greece). The selected EVOO samples were immediately placed in dark and cold storage (10 ± 2 °C) until further analyses. Regarding the extraction procedure, the method reported by Mohamed and co-authors [12] was applied. Briefly, three replicates from each sample (3 g each) were extracted in 3 mL of 80% methanol solution (*v/v*) (LC-MS grade, VWR, Milan, Italy) using a vortex mixer. The suspension was kept at room temperature for 30 min and then centrifuged at 6000× *g* for 10 min at 4 °C. The extracts were collected in 2 mL amber glass vials and then stored at −18 °C until analysis.

### 2.2. In Vitro Gastrointestinal Digestion

The in vitro gastrointestinal digestion was based on the protocol as detailed by Minekus et al. [24]. Simulated salivary fluid (SSF), simulated gastric fluid (SGF) and simulated intestinal fluid (SIF) were prepared following the scheme previously reported [24]. In particular, the method (scaled up for 500 µL of liquid sample) included an oral phase composed of SSF at pH 7.0, with salivary α-amylase (75 U/mL; from human saliva Type IX-A, Sigma-Aldrich Co. Milan, Italy). The oral step was run at 37 °C for 2 min. Then, the oral bolus samples were mixed (ratio 1:1) with the SGF at pH 3.0 containing porcine pepsin (2000 U/mL; P7000; Sigma-Aldrich Co. Milan, Italy). The gastric phase was carried out for 120 min at 37 °C. Gastric chyme was mixed (1:1) with the SIF at pH 7.0 containing pancreatin (100 U/mL; P1750; Sigma-Aldrich Co. Milan, Italy) and bile salts (10 mM; B8631; Sigma-Aldrich Co. Milan, Italy). The intestinal phase was carried out for 120 min at 37 °C. During the in vitro digestion, appropriate amounts of HCl (1 M) and NaOH (1 M) were added for pH adjustment. At selected time points (i.e., gastric and pancreatic end-phases), corresponding digestion sample tubes for each EVOO sample were cooled on ice to stop the reaction. The experiment was performed in triplicate (*n* = 3) and all the in vitro digestion steps were carried out in amber bottles in the dark.

### 2.3. UHPLC-QTOF Mass Spectrometry Analysis and Bioaccessibility

The polyphenol and sterol profiling of raw (i.e., prior to the in vitro digestion) EVOO samples, as well as after the different step of the in vitro static digestion method, was evaluated using a 1290 liquid chromatograph coupled with a G6550 mass spectrometer detector (from Agilent Technologies, Santa Clara, CA, USA), via a Dual JetStream Electrospray Ionization System (from Agilent Technologies, Santa Clara, CA, USA). In this regard, samples collected during the in vitro digestion process (i.e., after gastric and pancreatic phases) were centrifuged at 3000× *g* for 10 min at 4 °C. Thereafter, the oily phase was discarded (mainly containing undigested material), whereas the aqueous phase was retained (containing the end-products from the enzymatic hydrolysis) [25] and then filtered in vials using 0.22 μm cellulose syringe filters. The elution was performed using a mixture of water and acetonitrile (both LC-MS grade, VWR, Milan, Italy; both acidified with 0.1% formic acid) as a mobile phase and an Agilent Zorbax Eclipse-plus C18 column (100 × 2.1 mm, 1.8 μm). The gradient went from 6% acetonitrile to 94% acetonitrile in 32 min and the flow rate was 0.220 mL/min. The mass spectrometer worked in positive scan mode (100–1200 m/z), injecting 6 μL [26]. Source conditions were as follows: sheath gas nitrogen 10 L min^−1^ at 350 °C, drying gas 10 L min^−1^ at 280 °C, nebulizer pressure 60 psig, nozzle voltage 300 V, capillary voltage 3.5 kV. The sequence order was randomized, and three technical replicates were done. The software Agilent Profinder B.06 (from Agilent Technologies, Santa Clara, CA, USA) was used to elaborate mass raw features as previously reported [27]. In this regard, features were aligned, and monoisotopic accurate mass was combined with isotopic profile for compounds annotation, thus reaching a level 2 of confidence in annotation (i.e., putatively annotated compounds). A custom database was built and used as reference, considering the annotations provided in both “Food Database”, and “LIPID MAPS”. Besides, the monoisotopic mass accuracy tolerance was set to 5 ppm. Data pre-processing (mass and retention time alignment, compounds filtering) was carried out in an Agilent Profinder B.07, only those compounds identified within 100% of replications in at least one treatment were retained. This processed dataset was finally used for statistics and chemometrics. Thereafter, a calibration curve of cholesterol (Sigma grade, Sigma-Aldrich, S. Louis, MO, USA) reference solutions was used to estimate the total sterols content. Furthermore, pure methanolic standard solutions of individual phenolics (Extrasynthese, Lyon, France) at different concentrations were analyzed. The phenolic compounds detected by UHPLC-QTOF mass spectrometry were classified into phenolic class and sub-class, and then quantitative measurements were performed using the above-reported reference solutions, as previously reported [12,13]. A linear fitting (not forced to origin and not weighed) was built for quantitative purposes, then the abundance for each class as an equivalent of the reference compound within the class was expressed. Finally, polyphenols and sterols bioaccessibility during the in vitro digestion process was calculated according to Rocchetti et al. [28]:

*Bioaccessibility* (%) = (TC_A_/TC_B_) × 100

where TC_A_ is the total content of polyphenols or sterols in EVOO samples (mg/kg) after the in vitro digestion (considering each individual incubation phase), and TC_B_ is the total content of polyphenols or sterols in raw EVOO samples (mg/kg) prior to the in vitro digestion process.

### 2.4. Statistics and Chemometrics

Metabolomic data on the phytochemical profiles of different EVOO samples (during in vitro gastrointestinal digestion) were interpreted using the software Agilent Mass Profiler Professional B.12.06 (from Agilent Technologies, Santa Clara, CA, USA). The data treatment consisted in a filtering by abundance and by frequency, followed by a normalization at the 75th percentile and a baselining to the corresponding median in the dataset. Chemometrics and statistics were then carried out on this latter dataset. Firstly, unsupervised hierarchical cluster analysis (HCA; using ‘Wards’ as linkage rule with Squared Euclidean distance for similarity measure) was performed using a fold-change-based heat map. Thereafter, the metabolomic dataset was exported into SIMCA 13 (Umetrics, Malmo, Sweden), UV-scaled and elaborated for orthogonal projection to latent structures discriminant analysis (OPLS-DA) supervised modeling. Variation between the observation groups was separated into predictive (technical variation) and orthogonal (biological variation) components. The OPLS-DA model was checked for outliers (by using Hotelling’s T2 distribution) and then cross validated by means of CV-ANOVA (*p* < 0.01). Besides, a permutation testing was performed to exclude over-fitting after inspecting model parameters (goodness-of-fit, R^2^Y, and goodness-of-prediction, Q^2^Y). When considering the Q^2^Y prediction ability, a cut-off value > 0.5 was adopted as an acceptability threshold, as previously reported [29,30]. Thereafter, variable importance in projection (VIP analysis) was used to evaluate the importance of EVOO compounds and to select those with the highest discrimination potentials (VIP score > 1) during the in vitro gastrointestinal digestion process. The relationship among the variables of the OPLS-DA model was then summarized by means of a loading scatter plot. Finally, a naïve Bayesian biomarker identification of Mass Profiler Professional (i.e., the “find minimal entities” operated according to the forward selection algorithm) was carried out, targeting those 10 features able to better explain differences between treatments.

## 3. Results and Discussion

### 3.1. UHPLC-QTOF Screening of Polyphenols and Sterols in Raw EVOO Samples

The untargeted metabolomic approach based on UHPLC-QTOF mass spectrometry allowed us to putatively annotate 309 compounds, representative of the most important chemical classes of EVOO. In fact, we found an abundance of sterols (219 compounds), followed by polyphenols (67 compounds) and prenol lipids (mainly terpenoids). A detailed list containing each compound detected in our experimental conditions is reported in Supplementary Table S1, together with its composite mass spectrum.

According to the literature, the most important phenolics in olive oil can be classified into six categories, those being: cinnamic and benzoic acids, phenolic alcohols, secoiridoids, lignans, hydroxy-isochromans and flavonoids [7]. Looking more specifically to the individual content of the major and typical EVOO polyphenols (Supplementary Table S1), the highest content of both apigenin and luteolin (i.e., two of the main flavonoids) was found for the Leccino EVOO sample, being 1.31 and 0.36 mg Equivalents (Eq.)/kg, respectively. Regarding lignans, we found average contents of 0.33 mg Eq./kg for pinoresinol and 0.23 mg Eq./kg for 8-acetoxypinoresinol. In this regard, the Frantoio EVOO sample was the best source of pinoresinol (0.51 mg Eq./kg), whilst Leccino was abundant in 8-acetoxypinoresinol (0.41 mg Eq./kg). Another phenolic class characterizing EVOO is represented by phenolic acids. In our experimental conditions, the most abundant compound was cinnamic acid, recording an average content of 1.77 mg Eq./kg, whilst ferulic and 4-hydroxyphenylacetic acids showed lower contents (Supplementary Table S1). Overall, secoiridoids and phenolic alcohols are reported to be the most abundant compounds characterizing olive oil. In this regard, secoiridoids (including oleuropein and derivatives) are generated during the oil mechanical extraction process by endogenous β-glucosidases, which catalyze the hydrolysis of oleuropein, demethyloleuropein and ligstroside [7]. In olive oil, oleuropein is present in the aglycone form (i.e., oleuropein-aglycone). Accordingly, the average content of oleuropein-aglycone detected in the five EVOOs under investigation was 6.8-fold higher when compared to oleuropein (Supplementary Table S1), thus confirming what has been reported in the literature [31,32,33]. Interestingly, Leccino, Picual and Kalamon EVOOs were those samples characterized by the highest oleuropein-aglycone contents, being 17.2, 15.0 and 14.1 mg Eq./kg, respectively. Besides, in our experimental conditions, the dialdehydic form of decarboxymethyl ligstroside aglycone (*p*-HPEA-EDA) was the most abundant secoiridoid (Supplementary Table S1), recording higher values in the Frantoio (i.e., 90.1 mg Eq./kg) and Leccino (i.e., 84.5 mg Eq./kg) samples. Another important phenolic compound detected in EVOO is hydroxytyrosol. Looking to the individual semi-quantitative contents of hydroxytyrosol (belonging to phenolic alcohols) in the five EVOOs under investigation, we found an average content of 1.29 mg Eq./kg, with the higher value recorded in the Leccino sample (Supplementary Table S1). 

Regarding sterols, the untargeted metabolomics-based approach allowed us to annotate several classes of compounds, also revealing different semi-quantitative contents. In fact, several structure-related analogues of cholesterol were detected, (62 compounds), followed by sterol esters (36 compounds), stigmasterols and C24-ethyl derivatives (33 compounds) (Supplementary Table S1). Sterols are important lipids and are included in the unsaponifiable part of olive oil, with amounts ranging from 855 to 2185 mg/kg [7]. These compounds have been related to the quality of the olive oil and are broadly used for checking its genuineness. The main sterols present in olive oil are β-sitosterol, campesterol, stigmasterol, clerosterol, sitostanol and δ-5-avenasterol; therefore, our findings are in accordance with the literature [7]. Phytosterols are characterized by a chemical structure similar to cholesterol, but lacking an extra methyl or ethyl group, and are known to reduce the cholesterol absorption and thus, reduce its circulation levels [34]. Overall, significant differences were found considering the sterol content of the different EVOOs under investigation, ranging from 2020.9 (for Frantoio EVOO) up to 3706.9 mg Eq./kg (for Leccino EVOO). Finally, as it can be observed from Supplementary Table S1, other characteristic EVOO compounds were detected, including prenol lipids (mainly terpenes), amino acids and alkaloids. Looking at our data, significantly different phytochemical profiles could be gained in EVOOs from the cultivars considered. Notwithstanding, it must be carefully considered that the effect of environment (e.g., soil and climatic conditions, agronomic practices), processing and storage conditions may provide important contributions in terms of phytochemical profiles. These factors were not included in our experiments because they are out of the scope of this work.

### 3.2. Multivariate Discrimination of EVOO Samples after In Vitro Gastrointestinal Digestion

**Figure 1:**
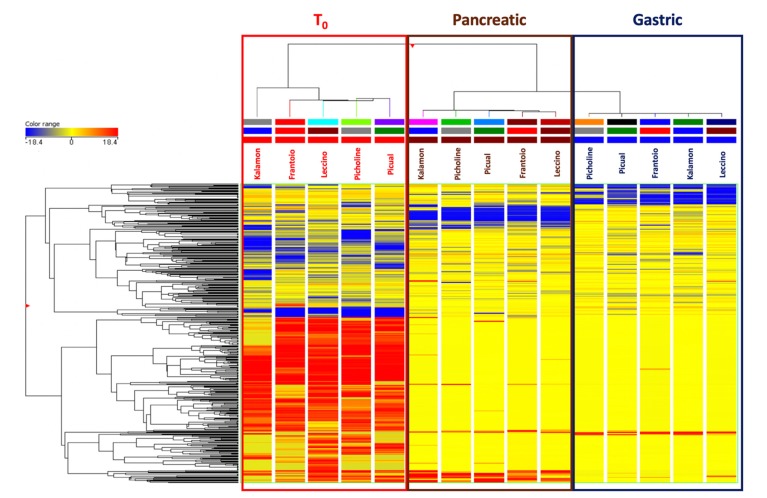
Unsupervised hierarchical cluster analysis (HCA) for the phytochemical profile of different Extra-Virgin Olive Oil (EVOO) samples T_0_ (raw; prior to digestion), gastric and pancreatic phases of in vitro gastrointestinal digestion. The cluster was built by considering the fold-change heat-map (similarity: Squared Euclidean; linkage rule: Ward). The color range represents the fold-change values used to build the heat-map.

In our experimental conditions, clear differences emerged between EVOOs prior to and after the in vitro gastrointestinal digestion process. In this regard, both the unsupervised HCA and the supervised OPLS-DA multivariate statistical approaches demonstrated the evolution of EVOO bioactive profile moving from T_0_ (raw samples) to gastric and then pancreatic phases of the simulated in vitro gastrointestinal digestion. In particular, the unsupervised HCA resulted in two main groups, with a first cluster including EVOO samples before digestion and a second cluster representing both digestive phases (i.e., gastric and pancreatic). The output of the HCA consisting on a heat-map built based on the fold-change values of each compound detected is reported in Figure 1.

**Figure 2:**
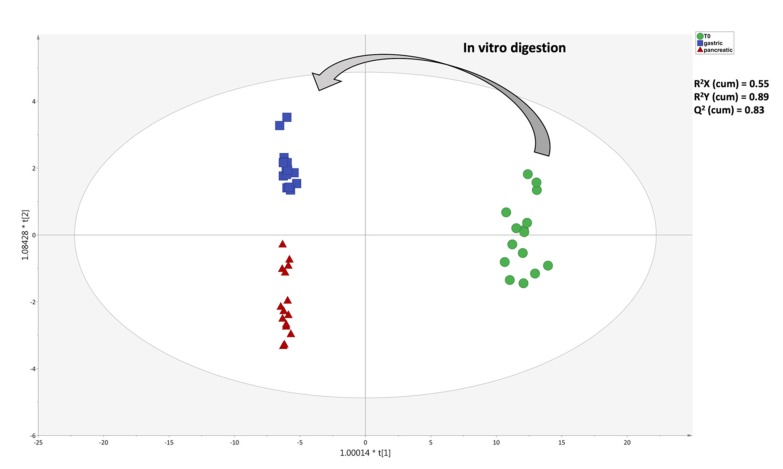
Orthogonal projection to latent structures discriminant analysis (OPLS-DA) score plot showing the modifications of the phytochemical composition of EVOO samples, moving from T_0_ (raw; prior to digestion) to gastric and pancreatic phases of in vitro gastrointestinal digestion.

The unsupervised naïve analysis highlighted a hierarchically stronger effect of digestion over cultivar differences, as expected. In detail, the raw EVOO samples clearly showed up-accumulated cluster of compounds when compared to in vitro digested samples; however, it was evident from the heat-map that both gastric and pancreatic phases of in vitro digestion were able to affect the phytochemical profile with a different incidence (Figure 1). Therefore, considering that the main differences during the in vitro gastrointestinal digestion were actually represented, the following multivariate supervised OPLS-DA was carried out aiming to investigate the contribution of each group of metabolites for discrimination purposes. This supervised approach allowed for pointing out differences across the phases of digestion irrespective from cultivars’ contributions. The OPLS-DA score plot built considering the modifications of EVOO samples during the in vitro digestion process is reported in Figure 2.

Looking at Figure 2, the OPLS-DA score plot evidenced a great degree of discrimination when considering the different EVOO samples, as imposed by the in vitro digestion process. Notably, the OPLS-DA model was characterized by more than acceptable goodness parameters, being R^2^X(cum) = 0.55, R^2^Y(cum) = 0.89 and Q^2^(cum) = 0.83. Therefore, both supervised and unsupervised multivariate statistics confirmed the suitability of untargeted metabolomics to highlight changes of bioactive compounds occurring during the in vitro gastrointestinal digestion process, thus sustaining a following investigation of the discriminant metabolites by means of the variable importance in projection (VIP) method. The latter provides the so-called VIP score (i.e., the contribution a variable makes to the OPLS model), allowing us to highlight the best markers of the distribution. In particular, the VIP score is calculated as a weighted sum of the squared correlations between the OPLS-DA components and the original variables. 

Table 1 reports those EVOO compounds possessing the higher degree of discrimination (VIP score > 1), grouped according to their chemical class, together with individual scores and standard error. Overall, 64 compounds were found to explain the changes in the phytochemical profiles during in vitro digestion of EVOO samples, with a marked abundance of flavonoids (15 compounds), followed by cholesterol and spirostanol analogues (accounting for 15 compounds). Interestingly, 10 compounds (i.e., peonidin, luteolin, pelargonidin, hispidulin, oleuropein, hydroxytyrosol, 4-hydroxybenzoic acid, 2α,7β,15β,18-tetraacetoxy-cholest-5-en-3α-ol, Nebrosteroid L and 6-O-(Glcb)-(25R)-5α-spirostan-3β,6α,23S-triol) were confirmed by naïve Bayesian analysis as the most discriminant compounds during the in vitro gastrointestinal digestion process. 

The VIP markers arising following the OPLS-DA model are not totally surprising; in this regard, flavonoids are reported to be not totally stable under the digestion condition [35,36,37]. This might be associated with the interaction between flavonoids and other components, such as digestive enzymes and/or to the conversion of polyphenols into other unknown or undetected compounds under dramatic pH changes (e.g., anthocyanins). It is also important to highlight that glycosidic forms of flavonoids and other phenolics underwent an in vitro digestion process and can be easily degraded under a mildly alkaline condition to form the corresponding aglycones [35,36,37]. Regarding the other VIP markers, both secoiridoids and phenolic alcohols were detected. In particular, oleuropein, oleuropein-aglycone and hydroxytyrosol were all included among the discriminant compounds, thus suggesting a clear impact of the in vitro digestion process on their initial content. Overall, the highest VIP scores were recorded for three compounds, namely 3-*O*-(alpha-L-rhamnopyranosyl)-3beta,14beta-dihydroxybufa-4,20,22-trienolide (a withanolide), 4-hydroxybenzoic acid (a phenolic acid) and 8-acetoxy-4’-methoxypinoresinol (a lignan), being 2.67, 2.12 and 1.64, respectively. These trends were confirmed by inspecting the VIP loading plot (Supplementary Table S1), where a clear modification of the phytochemical profiles was observed as a consequence of the in vitro digestion process, leading to a deep modification of those compounds characterizing the raw EVOO samples. In this regard, the VIP loading plot revealed that lower molecular weight phenolics (such as hydroxytyrosol and 4-hydroxybenzoic acid) were those markers better explaining the changes during gastric and pancreatic phases. 

### 3.3. Bioaccessibility and Modifications of Secoiridoids During In Vitro Gastrointestinal Digestion

Considering the modification trends revealed by both unsupervised and supervised statistics, it was evident that polyphenols and sterols were the classes of compounds most affected by the in vitro digestion process. Therefore, the % of bioaccessibility (focusing on classes of compounds) was calculated to point out the most stable classes under in vitro conditions. The bioaccessibility corresponds to the fraction of each compound released from the food matrix in the gastrointestinal tract, which then becomes available for absorption. A comprehensive overview of the different % bioaccessibility values following in vitro gastrointestinal digestion of EVOO samples is reported in Table 2.

As provided in the table, flavone equivalents were characterized by the lowest bioaccessibility values in all EVOO sample, and when considering both gastric and pancreatic phases of digestion. Regarding the other classes of compounds, anthocyanins experienced an average 10-fold reduction when moving from raw (undigested) to digested samples. Interestingly, Picholine was found to register a maximum % bioaccessibility in the pancreatic phase of 33%, definitely higher than other samples (on average: 12%). Lignans (quantified as matairesinol equivalents) were scarcely bioaccessible in both digestion phases, with maximum values (i.e., 22% and 18%) recorded for Kalamon and Leccino during the gastric phase. It is noteworthy that a moderate bioaccessibility of lignans could be pointed out by previous literature [38]. Similar trends were recorded for phenolic acids (quantified as ferulic acid equivalents). In this regard, Picual was characterized by the highest % bioaccessibility values in both phases, being 25% (gastric) and 21% (pancreatic) (Table 2). The most interesting bioaccessibility trends were recorded for other polyphenols (including secoiridoids and phenolic alcohols), quantified as tyrosol equivalents. In this regard, tyrosols were the most abundant class of phenolics in raw EVOO samples, ranging from 248.3 (Frantoio) up to 556.3 (Kalamon) mg Eq./kg. The tyrosols content was in strict agreement with previous works based on a detailed metabolomic profiling in different EVOO samples [12,13,39]. Thereafter, following the in vitro digestion process, relatively high % bioaccessibility values could observed following both gastric and pancreatic phases, recording average values of 53.2% and 65.2%, respectively. The trends observed in the % bioaccessibility of tyrosols were common for each EVOO sample, except for Kalamon (i.e., the sample showing the higher cumulative content before digestion). Therefore, according to our findings, it was possible to postulate that the % bioaccessibility values were quite similar over the different digestion phases, independently from the initial content (i.e., before digestion).

Regarding sterols, low bioaccessibility values were recorded when considering each EVOO sample, with a great impact of in vitro digestion on the initial contents, showing an average 10-fold reduction (Table 2). According to Reference [40], phytosterols are digested and absorbed primarily in the small intestine, due to hydrolysis of their lipophilic constituents by lipase, and then micellarized via bile salts. In particular, because of their excessive hydrophobicity, cholesterol and phytosterols require solubilization into intestinal mixed micelles, composed of bile acid salts and fatty acids. Besides, like other unsaturated lipids, phytosterols are prone to oxidation, giving rise to a group of compounds known as phytosterol oxidation products. Thus, sterol solubility represents one of the most important parameters to better understand their oxidative stability and bioaccessibility in the gut. In this regard, when affinity for the aqueous phase is considered, cholesterol still maintains the highest solubility, whilst campesterol always presents higher solubility than sitosterol and sitostanol derivatives. Therefore, starting from the previous considerations, it appears evident that sterol structure plays a pivotal role in the solubility and transfer of these compounds to the aqueous phase. In this regard, the increase in partitioning into the aqueous phase was found to be more rapid in the presence of pancreatic lipase [41].

**Figure 3:**
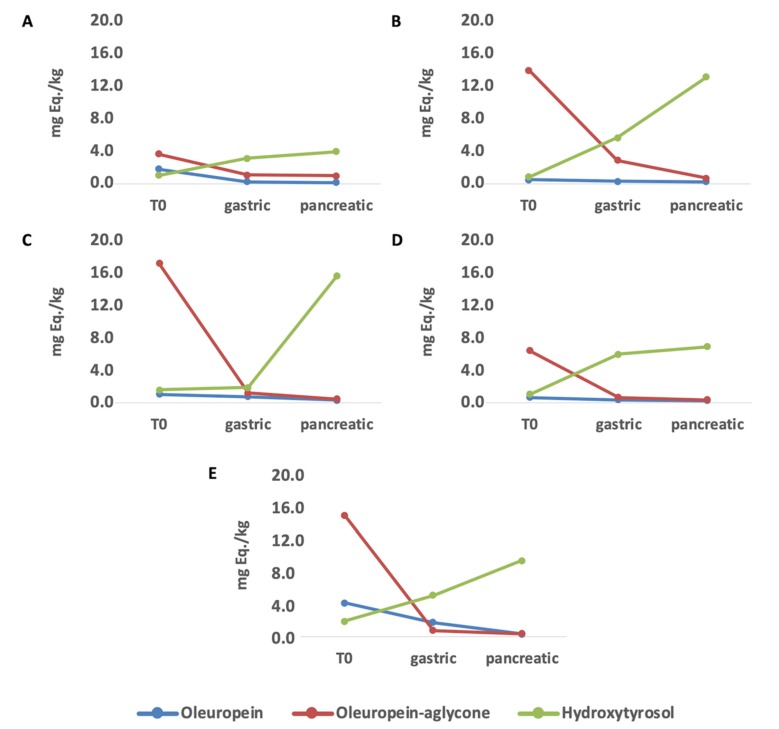
Main changes during in vitro gastrointestinal digestion involving oleuropein, oleuropein-aglycone and hydroxytyrosol in the different EVOO samples. (**A**) Frantoio, (**B**) Kalamon, (**C**) Leccino, (**D**) Picholine, (**E**) Picual.

Finally, the major changes involving oleuropein-aglycone (the major EVOO phenolic compound) during in vitro gastrointestinal digestion were investigated and are reported in Figure 3, considering each EVOO sample under investigation.

Overall, despite semi-quantitative differences between the different EVOO samples, the modification trends revealed a clear reduction of both oleuropein-aglycone and oleuropein over different digestion phases. These modifications were already evident during the gastric phase of in vitro digestion, thus confirming those highlighted by the VIP selection method following OPLS-DA modeling (Table 1). Interestingly, looking to hydroxytyrosol, an inverse trend was observed, with higher contents after 2 h of the pancreatic step for this phenolic alcohol. In particular, higher concentrations of hydroxytyrosol were observed for Leccino and Kalamon in the pancreatic phase, recording 15.7 and 13.2 mg Eq./kg, respectively. Hydroxytyrosol is a degradation product of oleuropein both in the olive fruit and in the human body. In this regard, the hydrolysis of oleuropein and its aglycone occurs after oil ingestion, by means of lipase activity, thus leading to the appearance of hydroxytyrosol [42]. According to the literature, there are no conclusive reports that clarify the pharmacokinetics of oleuropein, and it is reasonable to believe that only a small amount of unchanged oleuropein is able to reach the systemic circulation [15]. Besides, the biotransformation process of oleuropein and its aglycone is reported to be strictly dependent to the temperature, capable of shortening the enzymatic hydrolysis of oleuropein and chemical hydrolysis of oleuropein-aglycone in hydroxytyrosol [43]. Regarding the increasing trends observed in this work, it is conceivable that hydroxytyrosol is mainly derived from oleuropein-aglycone (showing higher content than oleuropein). In particular, oleuropein-aglycone can be converted under gastrointestinal conditions in two dialdehydes that are quite unstable, and in the lipid/water interface leading to the formation of the so-called transposed secoiridoid. This compound, under prolonged acid conditions and following the cleavage of the two ester groups, can lead to the formation of hydroxytyrosol [15].

## 4. Conclusions

Five commercial EVOO oils, representing cultivars characteristic of the Mediterranean basin, were investigated for their phytochemical profile before and following in vitro digestion, with a main focus on phenolic profile. Despite the fact that untargeted metabolomics allowed for depicting significant differences in terms of both phenolic content and phenolic profile across the cultivars, the changes imposed by in vitro digestion resulted to be hierarchically higher than cultivar effects. In particular, a relatively low bioaccessibility could be observed for most of the polyphenols moving towards gastric and then intestinal phases. Interestingly, although secoiridoids concentration declined during the digestion process, hydroxytyrosol concentration showed an opposite trend. In agreement with previous literature, this compound might play an important role in terms of phenolics intake coming from olive oil. Irrespectively from the effect of the cultivar considered, the present experiments suggest that the remarkable effect of digestion must be carefully considered when looking at the health-promoting effect of phenolics-rich plant foods. Nevertheless, our findings suggest the need for further dedicated experiments to confirm in vivo bioaccessibility of phenolics and the bioavailability of hydroxytyrosol.

## Figures and Tables

**Table 1:** VIP (variables importance in projection) markers following supervised OPLS-DA during in vitro gastrointestinal digestion of different EVOO samples. * = confirmed by naïve Bayesian analysis.

Class	VIP Marker (OPLS-DA)	VIP Score
*Alkaloids*	Dihydrocinchonine	1.09 ± 0.42
*Amino acids*	L-Tryptophan	1.10 ± 0.21
*Benzofurans*	Halleridone	1.37 ± 1.71
*Fatty acyls*	(Z)-3-Hexen-1-ol	1.16 ± 0.20
*Flavonoids*	Pelargonidin 3-*O*-glucoside	1.25 ± 0.22
	Peonidin ^*^	1.16 ± 0.20
	Apigenin	1.08 ± 0.34
	Luteolin ^*^	1.08 ± 0.31
	Pelargonidin ^*^	1.08 ± 0.30
	Chrysoeriol 7-*O*-glucoside	1.07 ± 0.43
	Cyanidin	1.07 ± 0.35
	Hispidulin ^*^	1.07 ± 0.30
	Luteolin 7-*O*-malonyl-glucoside	1.06 ± 0.43
	Delphinidin 3-*O*-arabinoside	1.06 ± 0.41
	Peonidin 3-*O*-rutinoside	1.04 ± 0.31
	Cyanidin 3-*O*-sambubioside 5-*O*-glucoside	1.04 ± 0.48
	Rhoifolin	1.03 ± 0.46
	Peonidin 3-*O*-(6’’-acetyl-glucoside)	1.01 ± 0.18
	Apigenin 6-*C*-glucoside	1.01 ± 0.23
*Lignans*	8-acetoxy-4’-methoxypinoresinol	1.64 ± 1.45
	(-)-Olivil	1.15 ± 0.50
	Secoisolariciresinol	1.08 ± 0.19
*Organooxygen compounds*	Salidroside	1.21 ± 0.09
	Hexanal	1.16 ± 0.20
*Other phenolics*	Oleuropein ^*^	1.26 ± 0.91
	3,4-dihydroxyphenylethanol-4-diglucoside	1.16 ± 0.15
	Oleuropein-aglycone	1.06 ± 0.73
	Hydroxytyrosol ^*^	1.05 ± 0.84
	Ligustroside	1.01 ± 0.23
*Phenolic acids*	4-hydroxybenzoic acid ^*^	2.12 ± 0.77
	Coumaric acid	1.11 ± 0.21
	Cinnamic acid	1.04 ± 0.24
*Prenol lipids*	(S)-Oleuropeic acid	1.26 ± 1.05
	Oleuroside	1.26 ± 0.92
	3β-Myrianthic acid	1.04 ± 0.24
*Pyridines and derivatives*	Pyridoxine	1.01 ± 0.22
*Bufanolides*	Hellebrigenin	1.08 ± 0.89
	Scillarenin	1.03 ± 0.25
*Cardanolides*	Cannogenin	1.11 ± 0.38
*Cholesterol analogues*	7-oxo-cholestenone	1.14 ± 0.19
	19-norcholestenone	1.11 ± 0.17
	Cholest-7-en-3β,5α,6β,9α-tetrol	1.10 ± 0.23
	24-northornasterol A	1.10 ± 0.38
	2α,7β,15β,18-tetraacetoxy-cholest-5-en-3α-ol ^*^	1.09 ± 0.21
	2-deoxy-20-hydroxy-5α-ecdysone 3-acetate	1.07 ± 0.47
	2-deoxyecdysone 22-phosphate	1.07 ± 0.29
	20-hydroxyecdysone	1.06 ± 0.24
	2-dehydroecdysone	1.04 ± 0.47
*Ergosterol derivatives*	Stoloniferone F	1.09 ± 0.28
	Typhasterol	1.04 ± 0.30
	Makisterone B	1.02 ± 0.29
	Nebrosteroid L ^*^	1.00 ± 0.34
*Spirostanol derivatives*	Episceptrumgenin	1.16 ± 0.19
	3-*O*-(Glcb1-3Glcb1-4(Rhaa1-2)Glcb)-(25R)-spirost-5-en-3β-ol	1.10 ± 0.25
	(23S,24R,25S)-23,24-dihydroxy-spirost-5-en-3β-yl-*O*-α-L-rhamnopyranosyl-(1-2)-β-D-glucopyranoside	1.08 ± 0.36
	Agavegenin A	1.08 ± 0.30
	6-*O*-(Glcb)-(25R)-5α-spirostan-3β,6α,23S-triol ^*^	1.04 ± 0.27
	3-*O*-(Galb)-(25R)-12-oxo-5α-spirostan-3β-ol	1.02 ± 0.31
*Stigmasterol derivatives*	Norselic acid A	1.07 ± 0.39
	Norselic acid E	1.06 ± 0.49
*Withanolide derivatives*	Proscillaridin A	2.67 ± 0.72
	Minabeolide-8	1.18 ± 0.03
	15β-Hydroxynicandrin B	1.01 ± 0.45
	Minabeolide-5	1.00 ± 0.49

**Table 2:** Semi-quantitative contents of polyphenols and sterols (expressed as mean value, *n* = 3) in EVOO samples prior to the in vitro digestion (T_0_), together with their changes during in vitro gastrointestinal digestion, considering both gastric and pancreatic phases. The % bioaccessibility value for each digestion phase is reported in round brackets.

Equivalents	EVOO	T_0_ (mg/kg)	Gastric Phase (mg/kg)	Pancreatic Phase (mg/kg)
*Cyanidin*	Frantoio	0.17	0.01 (8%)	0.02 (14%)
	Leccino	0.22	0.01 (4%)	0.02 (10%)
	Picholine	0.08	0.01 (12%)	0.03 (33%)
	Kalamon	0.20	0.01 (6%)	0.02 (10%)
	Picual	0.15	0.01 (8%)	0.02 (15%)
*Luteolin*	Frantoio	0.59	0.00 (1%)	0.01 (1%)
	Leccino	0.73	0.00 (0%)	0.01(1%)
	Picholine	0.30	0.00 (1%)	0.00(2%)
	Kalamon	0.58	0.00 (1%)	0.01(1%)
	Picual	0.46	0.01 (2%)	0.01(2%)
*Matairesinol*	Frantoio	11.83	0.77 (7%)	0.71 (6%)
	Leccino	5.30	0.98 (18%)	0.71 (13%)
	Picholine	6.72	0.75 (11%)	1.16 (17%)
	Kalamon	6.18	1.39 (22%)	1.13 (18%)
	Picual	8.80	0.67 (8%)	0.91 (10%)
*Tyrosol*	Frantoio	248.25	138.18 (56%)	189.81 (76%)
	Leccino	259.72	142.57 (55%)	201.86 (78%)
	Picholine	252.37	164.12 (65%)	196.46 (78%)
	Kalamon	556.28	244.72 (44%)	191.79 (34%)
	Picual	353.74	162.04 (46%)	212.56 (60%)
*Ferulic acid*	Frantoio	13.81	1.66 (12%)	1.38 (10%)
	Leccino	10.13	1.73 (17%)	1.84 (18%)
	Picholine	9.80	1.09 (11%)	1.29 (13%)
	Kalamon	9.20	1.46 (16%)	1.30 (14%)
	Picual	6.75	1.69 (25%)	1.43 (21%)
*Cholesterol*	Frantoio	2020.86	194.51 (10%)	221.22 (11%)
	Leccino	3706.94	135.82 (4%)	188.00 (5%)
	Picholine	3592.74	150.85 (4%)	263.46 (7%)
	Kalamon	2617.99	205.50 (8%)	248.45 (9%)
	Picual	3098.74	161.06 (5%)	271.07 (9%)

## Supplementary Materials

The following are available online at https://www.mdpi.com/2076-3921/9/4/302/s1: Table S1: dataset containing the compounds identified by UHPLC-QTOF-mass spectrometry, together with concentrations of typical EVOO compounds (prior to the in vitro digestion), OPLS-DA cross-validation parameters and VIP loadings plot.
